# The *NOD2* Single Nucleotide Polymorphism rs72796353 (IVS4+10 A>C) Is a Predictor for Perianal Fistulas in Patients with Crohn's Disease in the Absence of Other *NOD2* Mutations

**DOI:** 10.1371/journal.pone.0116044

**Published:** 2015-07-06

**Authors:** Fabian Schnitzler, Matthias Friedrich, Christiane Wolf, Johannes Stallhofer, Marianne Angelberger, Julia Diegelmann, Torsten Olszak, Cornelia Tillack, Florian Beigel, Burkhard Göke, Jürgen Glas, Peter Lohse, Stephan Brand

**Affiliations:** 1 Department of Medicine II-Grosshadern, Ludwig-Maximilians-University (LMU), Munich, Germany; 2 Department of Preventive Dentistry and Periodontology, LMU, Munich, Germany; 3 Max-Planck-Institute of Psychiatry, Biostatistics Group, Munich, Germany; 4 Department of Human Genetics, Rheinisch-Westfälische Technische Hochschule (RWTH) Aachen, Germany; 5 Department of Clinical Chemistry-Grosshadern, LMU, Munich, Germany; Indiana University School of Medicine, UNITED STATES

## Abstract

**Background:**

A previous study suggested an association of the single nucleotide polymorphism (SNP) rs72796353 (IVS4+10 A>C) in the *NOD2* gene with susceptibility to Crohn’s disease (CD). However, this finding has not been confirmed. Given that *NOD2* variants still represent the most important predictors for CD susceptibility and phenotype, we evaluated the association of rs72796353 with inflammatory bowel disease (IBD) susceptibility and the IBD phenotype.

**Methodology:**

Genomic DNA from 2256 Caucasians, including 1073 CD patients, 464 patients with ulcerative colitis (UC), and 719 healthy controls, was genotyped for the *NOD2* SNP rs72796353 and the three main CD-associated *NOD2* mutations rs2066844, rs2066845, and rs2066847. Subsequently, IBD association and genotype-phenotype analyses were conducted.

**Results:**

In contrast to the strong associations of the *NOD2* SNPs rs2066844 (p=3.51 x 10^-3^), rs2066845 (p=1.54 x 10^-2^), and rs2066847 (p=1.61 x 10^-20^) with CD susceptibility, no significant association of rs72796353 with CD or UC susceptibility was found. However, in CD patients without the three main CD-associated *NOD2* mutations, rs72796353 was significantly associated with the development of perianal fistulas (p=2.78 x 10^-7^, OR 5.27, [95% CI 2.75-10.12] vs. *NOD2* wild-type carriers).

**Conclusion/Significance:**

Currently, this study represents the largest genotype-phenotype analysis of the impact of the *NOD2* variant rs72796353 on the disease phenotype in IBD. Our data demonstrate that in CD patients the IVS4+10 A>C variant is strongly associated with the development of perianal fistulas. This association is particularly pronounced in patients who are not carriers of the three main CD-associated *NOD2* mutations, suggesting rs72796353 as additional genetic marker for the CD disease behaviour.

## Introduction

The two major forms of inflammatory bowel disease (IBD) Crohn’s disease (CD) and ulcerative colitis (UC) are characterized by a chronic and relapsing intestinal inflammation caused by a disrupted epithelial barrier integrity, probably due to an exaggerated inflammatory response to the luminal microbiota [1]. Epidemiological observations from IBD families, twin studies, cohort studies, and genome-wide association studies (GWAS) led to the identification of numerous IBD susceptibility genes [2],[3],[4]. Nucleotide-binding oligomerization domain 2 (*NOD2*, GeneID: 64127), the first CD gene identified, gave rise to the hypothesis that the host’s genetic background is one key factor that crucially influences the mucosal response to the luminal microbiota, and may lead to an aberrant, pathogenic immune response [5]. GWAS identified a total of 163 IBD susceptibility loci, including *IL23R*, *ATG16L1*, and *IRGM* [4,6–10]. Among these loci, the three *NOD2* mutations rs2066844, rs2066845, and rs2066847 still represent the strongest genetic predictors of CD susceptibility and phenotype [4].

In clinical practice, *NOD2* represents the most important genetic predictor of the CD disease course, which is heterogeneous and progresses differently among patients. Epidemiological data have shown that within 20 years, up to 90% of CD patients will experience either a penetrating or a fistulizing disease phenotype, often necessitating intensive medical treatment or surgery [11]. Early identification of a rapid progression of the disease would help to initiate an active treatment at an early disease stage to modulate the course of CD. In previous genotype-phenotype correlations, we and others could demonstrate a significant association of the three main *NOD2* mutations with ileal involvement, a stricturing phenotype and an early disease onset in CD patients [5,12–20].

In 2002, a French group published an analysis of the *NOD2* gene by direct DNA sequencing in 453 patients with CD, including 166 sporadic and 287 familial cases, 159 patients with ulcerative colitis (UC), and 103 healthy control subjects[13]. A total of 67 sequence variations were identified, and the three main NOD2 mutations (p.Arg702Trp, p.Gly908Arg, and p.Leu1007fsX1008) were confirmed to be independently associated with CD susceptibility. In addition, 27 rare variants were identified as potential disease-causing mutations, including the *NOD2* SNP rs72796353 (IVS4+10 A>C) [13].

In the study by Lesage et al. [13], the *NOD2* SNP rs72796353 was only found in CD patients, suggesting a potential role in CD development [13]. Apart from that, data on this SNP located in the intervening sequence of exon 4 are very limited [21,22] and there are no data analysing the phenotypic consequences of this SNP in IBD patients. Therefore, we aimed to analyse the association of the *NOD2* SNP rs72796353 regarding diseases susceptibility and disease phenotype in a large, well-characterized German IBD cohort (n = 2256).

## Patients and Methods

### Ethical Statement

All individuals gave their written, informed consent prior to the study. The study was approved by the local Ethics committee (Ludwig-Maximilians-University Munich) and adhered to the ethical principles for medical research involving human subjects of the Helsinki Declaration.

### Study population

A total of 2256 individuals were enrolled in this study, including a total of 1537 IBD patients of Caucasian origin (1073 CD patients and 464 UC patients) as well as 719 healthy, unrelated controls. All IBD patients were recruited from the University Hospital Munich-Grosshadern, while the 719 controls were recruited from the University Hospital Munich-Innenstadt. Patients with indeterminate colitis were excluded from the study. All individuals gave their written, informed consent prior to the study. Phenotypic parameters were collected blindly to the results of the genotype analysis and included demographic and clinical data (behaviour and anatomic location of IBD, disease-related complications, surgical or immunosuppressive therapy). Two senior gastroenterologists analysed the data which were recorded by patient charts analysis and a detailed questionnaire based on an interview at the time of enrolment. The diagnosis of CD and UC was based on established international guidelines, using endoscopic, radiological, and histopathological parameters [23]. CD patients were classified according to the Montreal classification [24] including age at diagnosis (A), location (L), and behaviour (B) of disease. In patients with UC, anatomic location was also assessed in accordance to the Montreal classification based on the criteria ulcerative proctitis (E1), left-sided UC (distal UC; E2), and extensive UC (pancolitis; E3). The demographic characteristics of the IBD study population are summarized in Table 1.

**Table 1:** Demographic characteristics of the IBD study population.

	Crohn’s disease	Ulcerative colitis	Controls
	n = 1073	n = 464	n = 719
**Gender**			
Male (%)	512 (47.7)	247 (53.2)	475 (66.1)
Female (%)	561 (52.3)	217 (46.8)	244 (33.9)
**Age** (yrs)			
Mean ± SD	40.4 ± 14.2	43.2 ± 15.4	47.4 ± 9.2
Range	(5–86)	(8–91)	(31–68)
**Body mass index**			
Mean ± SD	23.1 ± 4.2	23.9 ± 4.5	
Range	(13.2–40.8)	(15.2–53.6)	
**Age at diagnosis** (yrs)			
Mean ± SD	26.8 ± 12.0	30.7 ± 13.6	
Range	(2–78)	(4–81)	
**Disease duration** (yrs)			
Mean ± SD	14.6 ± 9.4	13.0 ± 8.7	
Range	(0–50)	(0–50)	
**Positive family history of IBD** (%)			
	117 (10.9)	40 (8.6)	

### DNA extraction and NOD2 genotyping

Genomic DNA was isolated from peripheral blood leukocytes and amplification of *NOD2* exons 4, 8 and 11 was performed by standard procedures. Primers used for PCR amplification of *NOD2* exons 4, 8, and 11 are listed in the S1 Table. Sequences were analysed on an ABI PRISM 377 DNA Sequencer (Applied Biosystems) using the Sequence Analysis program version 3.4.5 (Applied Biosystems). Control subject and patient sequence data were compared to the published *NOD2* sequence, and all differences were documented. Primers used for DNA sequence analysis of *NOD2* exons 4, 8, and 11 are listed in S2 Table.

### Statistical analyses

Each genetic marker was tested for Hardy-Weinberg equilibrium in the control population. Single-marker allelic tests were performed with Pearson’s χ2 test. All tests were two-tailed and p-values < 0.05 were considered significant. Odds ratios were calculated for the minor allele at each SNP. For evaluation of phenotypic consequences, we conducted logistic regression analyses. Data and haplotype analyses were done by using the Plink v1.07 software (http://pngu.mgh.harvard.edu/purcell/plink/[25] and R-2.4.1. (http://cran.r-project.org).

## Results

### The *NOD2* variant IVS4+10 A>C (rs72796353) is not significantly associated with susceptibility to Crohn’s disease or ulcerative colitis

Table 1 shows the demographic characteristics of the study population. A total of 1073 patients with CD, 464 patients with UC, and 719 controls with no history of IBD were included in the analysis. The median disease duration was 14.6 years for CD patients and 13.0 years for UC patients. 11% of the CD patients had a positive family history of IBD compared to 8% of the UC patients. There was no significant difference regarding the allele frequencies of the *NOD2* variant IVS4+10 A>C (rs72796353) in CD patients and controls (allele frequency of 3.17% in CD patients vs. 2.85% in controls (p = 0.588, OR 1.12 [0.75–1.65], Table 2), or UC patients and controls (allele frequency of 1.75% in patients with UC vs. 2.85% in controls (p = 0.081, OR 0.60 [0.33–1.07], S3 Table). Allele frequencies of the three main NOD2 mutations rs2066844, rs2066845 and rs2066847 in CD patients, UC patients and controls, respectively are given in S4 Table).

**Table 2:** CD phenotype stratified by the IVS4+10 A>C variant (rs72796353).

*NOD2* rs72796353	(1)	(2)	(1) vs. (2)	(1) vs. (2)	(1) vs. (2)
genotype status	AC/CC/-NOD2	*NOD2* wild-type	p-value	OR	95% CI
	n = 45	n = 593			
**Male sex (%)**					
	24 (53%)	271 (45%)	0.282	1.40	[0.76–2.56]
**Age at diagnosis** (yrs, based on median OR+CI for > median)					
Mean ± SD	25.2 ± 11.0	27.9 ± 12.1	0.632	1.16	[0.63–2.16]
Range	(10–58)	(4–78)			
**Disease duration** (yrs, based on median OR+CI for > median)					
Mean ± SD	17.3 ± 10.1	14.5 ± 9.3	0.163	0.65	[0.35–1.19]
Range	(3–39)	(0–42)			
**Body mass index** (kg/m², based on median OR+CI for > median)					
Mean ± SD	23.3 ± 4.5	23.3 ± 4.3	0.855	1.06	[0.55–2.08]
Range	(15.9–32.7)	(15.6–39.8)			
**Age at diagnosis**					
	(n = 41)	(n = 542)			
≤16 years (A1)	9 (22%)	73 (14%)	0.142	0.56	[0.26–1.21]
17–40 years (A2)	28 (68%)	396 (73%)	0.533	1.22	[0.65–2.28]
> 40 years (A3)	4 (10%)	73 (14%)	0.499	1.44	[0.50–4.13]
**Location**					
	(n = 45)	(n = 562)			
Terminal ileum (L1)	8 (18%)	114 (20%)	0.974	1.01	[0.46–2.24]
Colon (L2)	8 (18%)	87 (16%)	0.450	0.73	[0.33–1.64]
Ileocolon (L3)	28 (62%)	350 (62%)	0.195	0.66	[0.35–1.24]
Upper GI (L4)	1 (2%)	11 (2%)	0.902	0.88	[0.11–6.96]
Any ileal involvement (L1+L3)	36 (81%)	464 (83%)	0.066	0.50	[0.23–1.05]
**Behaviour** ^1^					
	(n = 43)	(n = 543)			
Non-stricturing, Non-penetrat. (B1)	6 (14%)	166 (30%)	0.129	1.80	[0.84–3.83]
Stricturing (B2)	10 (23%)	145 (27%)	0.747	0.90	[0.46–1.74]
Penetrating (B3)	27 (63%)	232 (43%)	**0.014**	0.45	[0.24–0.85]
**Use of immunosuppressive agents** ^2^					
	(n = 45)	(n = 561)	0.118	0.43	[0.15–1.24]
	40 (89%)	450 (80%)			
**Surgery because of CD** ^3^					
	(n = 41)	(n = 540)			
	29 (71%)	274 (51%)	**0.021**	0.44	[0.22–0.89]
**Fistulas**
	(n = 43)	(n = 543)			
	27 (63%)	232 (43%)	**0.014**	0.45	[0.24–0.85]
**Perianal fistulas**					
	19/43 (44%)	71/544 (13%)	**2.781 x 10^−7^**	0.19	[0.10–0.35]
**Stenosis**					
	(n = 41)	(n = 546)			
	24 (59%)	308 (56%)	0.841	0.94	[0.49–1.78]

Group (1), 43 CD patients heterozygous for the C allele of the rs72796353 variant and no further main *NOD2* mutant, and 2 patients homozygous for the C allele of the rs72796353 variant; group (2), CD patients without the IVS4+10 A>C variant and with no further main *NOD2* mutant (*NOD2* wild-type). ORs are based on the NOD2 wild-type group (e.g., NOD2 wild-type status is protective against perianal fistulas).

^1^ Disease behaviour was defined according to the Montreal classification. A stricturing disease phenotype was defined as presence of stenosis without penetrating disease. The diagnosis of stenosis was made surgically, endoscopically, or radiologically (using MR enteroclysis).

^2^ Immunosuppressive agents included azathioprine, 6-mercaptopurine, methotrexate, infliximab, and/or adalimumab.

^3^ Only surgery related to CD-specific problems (e.g., ileocecal resection, fistulectomy, colectomy, ileostomy) was included.

### The *NOD2* variant IVS4+10 A>C is significantly associated with the development of perianal fistulas

In a next step, we analyzed if the *NOD2* variant IVS4+10 A>C is associated with a specific CD phenotype. Table 2 shows the phenotypic characteristics of CD patients carrying the IVS4+10 A>C variant (AC/CC/-NOD2 group) compared to CD patients carrying no NOD2 variant (none of the three main variants and no *NOD2* IVS4+10 variant [13,26]; defined as *NOD2* “wild-type” (WT)). No significant differences were seen for age at diagnosis, disease duration, body mass index, use of immunosuppression, and disease location (Table 2).

More CD patients carrying the *NOD2* variant IVS4+10 A>C suffered from penetrating CD behaviour compared to patients with the *NOD2* WT (p = 0.014, OR 0.45 [0.24–0.85], Table 2). Moreover, the *NOD2* variant IVS4+10 A>C (rs72796353) was highly associated with the development of perianal fistulas in these patients (p = 2.78 x 10^−7^, OR 5.27 [2.75–10.12], OR based on the NOD2 IVS4+10 A>C carriers; OR 0.19 [0.10–0.35], OR based on the NOD2 wildtype group; Table 2). In addition, the number of patients requiring CD-related surgery was significantly higher in the AC/CC/-NOD2 group than in CD patients with the *NOD2* WT (p = 0.022) whereas there were no significant differences seen between the two groups regarding stricturing disease behaviour (Table 2).

### The predictive power of the IVS4+10 A>C variant for fistulizing CD disease behaviour is significantly higher in the absence of other *NOD2* mutations

To analyze potential gene-dosage effects of the IVS4+10 A>C (rs72796353) variant in combination with the main CD-associated NOD2 mutants, p.Arg702Trp, p.Gly908Arg, and p.Leu1007fsX1008 (rs2066844, rs2066845, and rs2066847), we compared rs72796353 minor (C) allele carriers (n = 66) with wild-type (AA) patients (n = 1007, S5 Table).

There were no significant differences between the two groups regarding gender, age at diagnosis, disease duration, BMI, use of immunomodulators, stricturing disease behaviour and CD-related surgery (S5 Table). However, significantly more minor allele carriers had perianal fistulas, compared to wild-type subjects (33.4% vs. 12.5% with perianal fistulas, p = 6.031 x 10^−6^, S5 Table).

Interestingly, more rs72796353 minor allele carriers with no further *NOD2* mutant (AC/CC/–NOD2 group) suffered from penetrating disease than carriers of the minor allele with an additional main *NOD2* mutant (AC/+NOD2 group, 62.8% vs. 40%, p = 0.043, OR 0.32 [0.11–0.97]). In addition, more CD patients in the AC/CC/–NOD2 group had perianal fistulas compared to patients in the AC/+NOD2 group (44% vs. 10%, p = 0.016, S6 Table).

## Discussion

Since the identification of *NOD2* as the first CD susceptibility gene in 2001, the three main NOD2 mutations p.Arg702Trp, p.Gly908Arg, and p.Leu1007fsX1008 still represent the most strongly CD-associated variants [4,27,28]. In a study of Lesage et al. [13], the rare *NOD2* variant IVS4+10 A>C (rs72796353) was only found in CD, suggesting its suitability as a predictor for CD susceptibility.

In our cohort of 1073 well-phenotyped CD patients, we could demonstrate that the *NOD2* variant IVS4+10 A>C is indeed a strong predictor for perianal fistulas in CD patients. This predictive power is significantly enhanced in patients carrying no other *NOD2* mutations, emphasizing its applicability for disease course prediction in the absence of the well-established three main *NOD2* mutants. However, we could not confirm the association between the *NOD2* variant rs72796353 and CD susceptibility as suggested by the study of Lesage et al.[13].

In clinical practice, detection of the three main *NOD2* mutants has a great impact on disease course prediction and is used in addition to clinical, endoscopic and radiological findings for decisions regarding therapy [14,15,18,19,26,29]. Although the potential disease-causing effects of several *NOD2* variants were already investigated in functional assays on the protein level, the effects of the IVS4+10 A>C variant rs72796353 have not yet been addressed in functional investigations [30]. Lying close to the exon/intron boundary of exon 4, this SNP might influence the length of the encoded protein by creating an alternative splice site. To analyze the role of the IVS4+10 A>C variant in IBD risk and phenotype, we analyzed this SNP in a large German IBD patient cohort, thereby confirming that this *NOD2* variant is more frequently present in CD patients than in UC patients. Interestingly, only a few genetic studies investigated this SNP in patients with IBD (S7 Table). In most studies, no significant difference was seen between CD patients and controls, whereas a French study [13] found this variant in CD patients but not in the control group (S7 Table). Our results were in accordance to the allele frequencies determined in the HAPMAP project (S7 Table), confirming the predictive power of our data set.

Regarding *NOD2* haplotype associations, a recently published meta-analysis of 49 genetic studies investigated the association between the risk of complicated CD conferred by the three main mutants Arg702Trp, Gly908Arg, and p.Leu1007fsX1008 [19]. A total of 8.893 patients with CD were included in the analysis, 2.897 of whom had *NOD2* mutations [19]. With the presence of a single *NOD2* mutant, the risk of complicated disease was increased by 8%. In compound heterozygous and homozygous carriers, the risk increase was 41% [19]. The surgery risk increased by 58% with any of the three main *NOD2* mutants, whereas the risk of perianal disease was not influenced [19]. These observations were recently confirmed by a Belgian single center study [18]. Importantly, we could demonstrate that the minor C allele of rs72796353 is highly associated with perianal disease in our cohort, strongly suggesting that this *NOD2* variant is a genetic marker to detect CD patients with a high risk of developing perianal fistulas.

Several years ago, we and others [14,15] suggested a targeted, early-onset intensive therapy for high-risk CD patients being homozygous carriers of the NOD2 frameshift mutation p.Leu1007fsX1008 [14,15]. The recently published studies confirmed these recommendations in high-risk patients with two *NOD2* mutations [18,19]. Likewise, rs72796353 could serve as a predictor for perianal disease in CD patients in the absence of other *NOD2* mutations, thereby increasing the set of reliable genetic markers for early disease course prediction, although larger and prospective studies are needed to confirm the predictive power of rs72796353 in daily clinical practice.

In conclusion, there was no significant disease association of the *NOD2* variant IVS4+10 A>C with CD and UC. However, in the absence of other *NOD2* mutations, the *NOD2* variant IVS4+10 A>C was significantly associated with perianal fistulas in CD patients, suggesting the IVS4+10 A>C variant as a novel predictor for perianal disease in CD.

## Supporting Information

S1 Table Primers used for PCR amplification of *NOD2* exons 4, 8, and 11. (DOC)

S2 Table Primers used for DNA sequence analysis of *NOD2* exons 4, 8, and 11. (DOC)

S3 Table Given are minor allele frequencies (MAF) of the *NOD2* variant rs72796353 (IVS4+10 A>C) in patients with Crohn’s disease, ulcerative colitis as well as in controls. Minor allele frequencies, allelic test *P*-values, and odds ratios (OR, shown for the minor allele) with 95% confidence intervals (CI) are depicted for both the CD and UC case-control cohorts.
(DOC)

S4 Table Given are allele frequencies of the three main NOD2 mutations, rs2066844, rs2066845, and rs2066847 in patients with Crohn’s disease and, ulcerative colitis as well as in controls. Minor allele frequencies (MAF), allelic test *P*-values, and odds ratios (OR, shown for the minor allele) with 95% confidence intervals (CI) are depicted for both the CD and UC case-control cohorts. Details on the phenotypes of a subgroup of these patients were reported in previous studies [17, 18].
(DOC)

S5 Table Association between the rs72796353 genotype and CD disease characteristics based on the Montreal classification [27]. For each variable, the number of patients included is given. ^1^Disease behaviour was defined according to the Montreal classification [27]. A stricturing disease phenotype was defined as presence of stenosis without penetrating disease. ORs (odds ratios) are shown for the AA allele. Diagnosis of stenoses was made surgically, endoscopically, or radiologically (using MR enteroclysis).^2^ Immunosuppressive agents included azathioprine, 6-mercaptopurine, methotrexate, infliximab, and/or adalimumab. ^3^ Only surgery related to CD-specific problems (e.g., ileocecal resection, fistulectomy, colectomy, ileostomy) was included.
(DOC)

S6 Table Phenotype stratified by genotype in CD patients carrying the SNP rs72796353 plus one of the main *NOD2* mutations (AC/+NOD2) and in CD patients carrying the SNP rs72796353 without one of the main *NOD2* mutations (AC/-NOD2). For each variable, the number of patients included is given. ^1^Disease behaviour was defined according to the Montreal classification [27]. A stricturing disease phenotype was defined as presence of stenosis without penetrating disease. The diagnosis of stenosis was made surgically, endoscopically, or radiologically (using MR enteroclysis).^2^ Immunosuppressive agents included azathioprine, 6-mercaptopurine, methotrexate, infliximab, and/or adalimumab. ^3^ Only surgery related to CD-specific problems (e.g., ileocecal resection, fistulectomy, colectomy, ileostomy) was included.
(DOC)

S7 Table Comparison of the observed allele frequencies of rs72796353 with data published in the literature (Fishers exact test). C allele frequencies in patients with CD were similar to the data reported by the HAPMAP project. Interestingly, they were significantly different compared to those published by Lesage et al. [16] and Tukel et al. [24] In the French study, rs72796353 was not found in controls, whereas Tukel et al. [24] observed significant lower allele frequencies in Jewish families compared to our German population.
(DOC)
