# Supplementary material for: The NOD2 Single Nucleotide Polymorphism rs72796353 (IVS4+10 A>C) Is a Predictor for Perianal Fistulas in Patients with Crohn's Disease in the Absence of Other NOD2 Mutations
Source: PLoS One. 2015 Jul 6;10(7):e0116044. doi: 10.1371/journal.pone.0116044 (PMC4493062; doi:10.1371/journal.pone.0116044)
Supplement: S1 Table — (DOC) [file pone.0116044.s001.doc]

| Exon | Primer | | Size of product  (bp) |
| --- | --- | --- | --- |
|  | forward | reverse |  |
| 4 | 5′- TTAGGTCCCGTCTTCACCATG -3′ | 5′- CTCCCACACTTAGCCTTGATGG -3′ | 1936 |
| 8 | 5′- GGAGGAGGACTGTTAGTTCATGTCTAG -3′ | 5′- CTCCTCCCTCTTCACCTGATCTC -3′ | 223 |
| 11 | 5′- GACAGGTGGGCTTCAGTAGACTG –3′ | 5′- GATCCTCAAAATTCTGCCATTCC -3′ | 293 |

**Supplemental table S1.** Primers used for PCR amplification of *NOD2* exons 4, 8, and 11.
