# Supplementary material for: The NOD2 Single Nucleotide Polymorphism rs72796353 (IVS4+10 A>C) Is a Predictor for Perianal Fistulas in Patients with Crohn's Disease in the Absence of Other NOD2 Mutations
Source: PLoS One. 2015 Jul 6;10(7):e0116044. doi: 10.1371/journal.pone.0116044 (PMC4493062; doi:10.1371/journal.pone.0116044)
Supplement: S2 Table — (DOC) [file pone.0116044.s002.doc]

| Exon | Primer | | orientation |
| --- | --- | --- | --- |
| 4.I | 5′- TGGTTTGGCCATGCACTG -3′ | forward | |
| 4.II | 5′- GGCAAGACTTCCAGGAATTTCTC -3′ | forward | |
| 4.III | 5′- ATCGAGCTGTACCTGAGGAAGC -3′ | forward | |
| 4.IV | 5′- GGGATGGAGTGGAAGTGCTTG –3′ | reverse | |
| 4.V | 5′- CTCCCACACTTAGCCTTGATGG -3′ | reverse | |
| 8 | 5′- GGAGGAGGACTGTTAGTTCATGTCTAG -3′ | forward | |
| 11 | 5′- GACAGGTGGGCTTCAGTAGACTG –3′ | forward | |

**Supplemental table S2.** Primers used for DNA sequence analysis of *NOD2* exons 4, 8, and 11.
