# Supplementary material for: The NOD2 Single Nucleotide Polymorphism rs72796353 (IVS4+10 A>C) Is a Predictor for Perianal Fistulas in Patients with Crohn's Disease in the Absence of Other NOD2 Mutations
Source: PLoS One. 2015 Jul 6;10(7):e0116044. doi: 10.1371/journal.pone.0116044 (PMC4493062; doi:10.1371/journal.pone.0116044)
Supplement: S3 Table — Minor allele frequencies, allelic test P-values, and odds ratios (OR, shown for the minor allele) with 95% confidence intervals (CI) are depicted for both the CD and UC case-control cohorts. (DOC) [file pone.0116044.s003.doc]

| **Gene marker** | **Minor** | **Crohn’s disease** | | | **Ulcerative colitis** | | | **Controls** |
| --- | --- | --- | --- | --- | --- | --- | --- | --- |
|  | **allele** | n=1073 | | | n=464 | | | n=719 |
|  |  | **MAF (%)** | **p value** | **OR [95 % CI]** | **MAF (%)** | **p value** | **OR [95 % CI]** | **MAF (%)** |
| rs72796353 | | | | | | | | |
| IVS4+10 A>C | C | 3.17 | 0.587 | 1.12 [0.75-1.65] | 1.75 | 0.081 | 0.60 [0.33-1.07] | 2.85 |

**Supplemental table S3.** Given are minor allele frequencies (MAF) of the *NOD2* variant rs72796353 (IVS4+10 A>C) in patients with Crohn’s disease, ulcerative colitis as well as in controls. Minor allele frequencies, allelic test *P*-values, and odds ratios (OR, shown for the minor allele) with 95% confidence intervals (CI) are depicted for both the CD and UC case-control cohorts.
