# Supplementary material for: The NOD2 Single Nucleotide Polymorphism rs72796353 (IVS4+10 A>C) Is a Predictor for Perianal Fistulas in Patients with Crohn's Disease in the Absence of Other NOD2 Mutations
Source: PLoS One. 2015 Jul 6;10(7):e0116044. doi: 10.1371/journal.pone.0116044 (PMC4493062; doi:10.1371/journal.pone.0116044)
Supplement: S4 Table — Minor allele frequencies (MAF), allelic test P-values, and odds ratios (OR, shown for the minor allele) with 95% confidence intervals (CI) are depicted for both the CD and UC case-control cohorts. Details on the phenotypes of a subgroup of these patients were reported in previous studies [17, 18]. (DOC) [file pone.0116044.s004.doc]

| **Gene marker** | **Minor** | **Crohn’s disease** | | | **Ulcerative colitis** | | | **Controls** |
| --- | --- | --- | --- | --- | --- | --- | --- | --- |
|  | **allele** | n=1073 | | | n=464 | | | n=719 |
|  |  | **MAF (%)** | **p value** | **OR [95 % CI]** | **MAF (%)** | **p value** | **OR [95 % CI]** | **MAF (%)** |
| rs2066844 |  |  |  |  |  |  |  |  |
| p.Arg702Trp | T | 8.0 | **3.51 x 10^-3^** | 1.63 [1.17-2.28] | 3.88 | 0.217 | 0.76 [0.49-1.18] | 5.06 |
| rs2066845 |  |  |  |  |  |  |  |  |
| p.Gly908Arg | C | 3.87 | **1.54 x 10^-2^** | 1.83 [1.11-2.99] | 1.62 | 0.394 | 0.75 [0.38-1.47] | 2.15 |
| rs2066847 |  |  |  |  |  |  |  |  |
| p.Leu1007fsX1008 | insC | 14.3 | **1.61 x 10^-20^** | 5.59 [3.73-8.37] | 2.80 | 0.896 | 0.96 [0.55-1.70] | 2.90 |

**Supplemental table S4.** Given are allele frequencies of the three main NOD2 mutations, rs2066844, rs2066845, and rs2066847 in patients with Crohn’s disease and, ulcerative colitis as well as in controls. Minor allele frequencies (MAF), allelic test *P*-values, and odds ratios (OR, shown for the minor allele) with 95% confidence intervals (CI) are depicted for both the CD and UC case-control cohorts. Details on the phenotypes of a subgroup of these patients were reported in previous studies [17, 18].
