# Supplementary material for: The NOD2 Single Nucleotide Polymorphism rs72796353 (IVS4+10 A>C) Is a Predictor for Perianal Fistulas in Patients with Crohn's Disease in the Absence of Other NOD2 Mutations
Source: PLoS One. 2015 Jul 6;10(7):e0116044. doi: 10.1371/journal.pone.0116044 (PMC4493062; doi:10.1371/journal.pone.0116044)
Supplement: S5 Table — For each variable, the number of patients included is given. 1Disease behaviour was defined according to the Montreal classification [27]. A stricturing disease phenotype was defined as presence of stenosis without penetrating disease. ORs (odds ratios) are shown for the AA allele. Diagnosis of stenoses was made surgically, endoscopically, or radiologically (using MR enteroclysis).2 Immunosuppressive agents included azathioprine, 6-mercaptopurine, methotrexate, infliximab, and/or adalimumab. 3 Only surgery related to CD-specific problems (e.g., ileocecal resection, fistulectomy, colectomy, ileostomy) was included. (DOC) [file pone.0116044.s005.doc]

| ***NOD2* rs72796353** | | | | | | |
| --- | --- | --- | --- | --- | --- | --- |
|  | **(1)** | | **(2)** | **(1) vs. (2)** | **(1) vs. (2)** | **(1) vs. (2)** |
| **genotype status** | **AA** | | **AC/CC** | **p-value** | **OR** | **95% CI** |
|  | n=1007 | | n=66 |  |  |  |
| **Male sex** | | | | | | |
|  | 481 (47.4%) | | 35 (53%) | 0.373 | 1.25 | [0.76-2.07] |
| **Age at diagnosis** (years, based on median OR+CI for > median) | | | | | | |
| Mean ± SD | 27.0 ± 12.1 | | 24.1 ± 10.2 | 0.325 | 1.29 | [0.77-2.16] |
| Range | (2-78) | | (9-58) |  |  |  |
| **Disease duration** (years, based on median OR+CI for > median) | | | | | | |
| Mean ± SD | 14.6 ± 9.3 | | 15.2 ± 10.3 | 0.508 | 1.20 | [0.70-2.04] |
| Range | (0-50) | | (0-39) |  |  |  |
| **Body mass index** (kg/m², based on median OR+CI for > median) | | | | | | |
| Mean ± SD | 23.2 ± 4.3 | | 22.6 ± 4.3 | 0.192 | 1.47 | [0.82-2.62] |
| Range | (13.2-40.8) | | (15.9-32.7) |  |  |  |
| **Age at diagnosis** | | | | | | |
|  | (n=935) | | (n=62) |  |  |  |
| ≤16 years (A1) | 146 (15.6%) | | 14 (23%) | 0.141 | 0.63 | [0.34-1.17] |
| 17-40 years (A2) | 670 (71.6%) | | 44 (71%) | 0.828 | 1.06 | [0.63-1.79] |
| > 40 years (A3) | 119 (12.7%) | | 4 (6%) | 0.164 | 2.08 | [0.74-5.81] |
| **Location** | | | | | | |
|  | | (n=959) | (n=64) |  |  |  |
| Terminal ileum (L1) | | 210 (22.0%) | 12 (18.8%) | 0.550 | 1.23 | [0.63-2.39] |
| Colon (L2) | | 123 (13.0%) | 9 (14.0%) | 0.613 | 0.83 | [0.40-1.72] |
| Ileocolon (L3) | | 613 (64.0%) | 44 (68.8%) | 0.125 | 0.67 | [0.40-1.12] |
| Upper GI (L4) | | 13 (1.4%) | 1 (1.6%) | 0.057 | 2.71 | [0.97-7.57] |
| Any ileal involvement  (L1+L3) | | 654 (85.8%) | 56 (87.5%) | 0.079 | 0.57 | [0.31-1.07] |
| **Behaviour** ^1^ | | | | | | |
|  | | (n=934) | (n=63) |  |  |  |
| Non-stricturing, Non-penetrat. (B1) | | 262 (28.0%) | 13 (20.6%) | 0.631 | 1.15 | [0.65-2.04] |
| Stricturing (B2) | | 254 (27.2%) | 16 (25.4%) | 0.986 | 0.10 | [0.57-1.73] |
| Penetrating (B3) | | 418 (44.6%) | 34 (54.0%) | 0.171 | 0.70 | [0.42-1.17] |
| **Use of immunosuppressive agents** ^2^ | | | | | | |
|  | | (n=945) | (n=66) |  |  |  |
|  | | 769 (81.4%) | 56 (84.8%) | 0.338 | 0.70 | [0.34-1.45] |
| **Surgery because of CD** ^3^ | | | | | | |
|  | | (n=917) | (n=61) |  |  |  |
|  | | 430 (56.2%) | 41 (67.0%) | 0.136 | 0.66 | [0.39-1.14] |
| **Fistulas** | | | | | | |
|  | | (n=928) | (n=63) |  |  |  |
|  | | 418 (45.0%) | 34 (54.0%) | 0.171 | 0.70 | [0.42-1.17] |
| **Perianal fistulas** | | | | | | |
|  | | 116/928 (12.5%) | 21/63 (33.4%) | **6.031x10^-6^** | 0.28 | [0.16-0.48] |
| **Stenosis** | | | | | | |
|  | | (n=930) | (n=59) |  |  |  |
|  | | 561 (60.3%) | 37 (62.7%) | 0.840 | 1.06 | [0.62-1.79] |

**Supplemental table S5.** Association between the rs72796353 genotype and CD disease characteristics based on the Montreal classification [27]. For each variable, the number of patients included is given. ^1^Disease behaviour was defined according to the Montreal classification [27]. A stricturing disease phenotype was defined as presence of stenosis without penetrating disease. OR (odds ratio), shown for the AA allele The diagnosis of stenoses was made surgically, endoscopically, or radiologically (using MR enteroclysis).^2^ Immunosuppressive agents included azathioprine, 6-mercaptopurine, methotrexate, infliximab, and/or adalimumab. ^3^ Only surgery related to CD-specific problems (e.g., ileocecal resection, fistulectomy, colectomy, ileostomy) was included.
