# Supplementary material for: The NOD2 Single Nucleotide Polymorphism rs72796353 (IVS4+10 A>C) Is a Predictor for Perianal Fistulas in Patients with Crohn's Disease in the Absence of Other NOD2 Mutations
Source: PLoS One. 2015 Jul 6;10(7):e0116044. doi: 10.1371/journal.pone.0116044 (PMC4493062; doi:10.1371/journal.pone.0116044)
Supplement: S6 Table — For each variable, the number of patients included is given. 1Disease behaviour was defined according to the Montreal classification [27]. A stricturing disease phenotype was defined as presence of stenosis without penetrating disease. The diagnosis of stenosis was made surgically, endoscopically, or radiologically (using MR enteroclysis).2 Immunosuppressive agents included azathioprine, 6-mercaptopurine, methotrexate, infliximab, and/or adalimumab. 3 Only surgery related to CD-specific problems (e.g., ileocecal resection, fistulectomy, colectomy, ileostomy) was included. (DOC) [file pone.0116044.s006.doc]

| ***NOD2* rs72796353** | | | | | | |
| --- | --- | --- | --- | --- | --- | --- |
|  | **(1)** | | **(2)** | **(1) vs. (2)** | **(1) vs. (2)** | **(1) vs. (2)** |
| **genotype status** | **AC/+NOD2** | | **AC/CC/–NOD2** | **p-value** | **OR** | **95% CI** |
|  | n=21 | | n=45 |  |  |  |
| **Male sex** | | | | | | |
|  | 11 (52.4%) | | 24 (53.0%) | 0.942 | 1.04 | [0.37-2.93] |
| **Age at diagnosis** (years, based on median OR+CI for > median) | | | | | | |
| Mean ± SD | 22.2 ± 8.0 | | 25.1 ± 11.1 | 0.290 | 0.55 | [0.18-1.67] |
| Range | (9-40) | | (10-58) |  |  |  |
| **Disease duration** (years, based on median OR+CI for > median) | | | | | | |
| Mean ± SD | 9.8 ± 9.1 | | 17.3 ± 10.1 | **0.009** | 0.06 | [0.01-0.51] |
| Range | (0-38) | | (3-39) |  |  |  |
| **Body mass index** (kg/m², based on median OR+CI for > median) | | | | | | |
| Mean ± SD | 21.1 ± 3.3 | | 23.3 ± 4.5 | 0.072 | 0.23 | [0.05-1.14] |
| Range | (17.8-29.1) | | (15.9-32.7) |  |  |  |
| **Age at diagnosis** | | | | | | |
|  | (n=21) | | (n=42) |  |  |  |
| ≤16 years (A1) | 5 (25%) | | 9 (21.4%) | 0.725 | 1.25 | [0.36-4.33] |
| 17-40 years (A2) | 15 (75%) | | 29 (69.1%) | 0.466 | 1.52 | [0.49-4.66] |
| > 40 years (A3) | 0 (0%) | | 4 (9.5%) | 0.994 | 3.26x10^-08^ | [0-Inf] |
| **Location** | | | | | | |
|  | | (n=21) | (n=45) |  |  |  |
| Terminal ileum (L1) | | 4 (19%) | 8 (17.8%) | 0.723 | 0.77 | [0.18-3.26] |
| Colon (L2) | | 1 (5%) | 8 (17.8%) | 0.182 | 0.23 | [0.03-1.98] |
| Ileocolon (L3) | | 16 (76%) | 28 (62.2%) | 0.727 | 1.21 | [0.41-3.61] |
| Upper GI (L4) | | 3 (14%) | 1 (2.2%) | 0.094 | 7.33 | [0.71-75.27] |
| Any ileal involvement  (L1+L3) | | 20 (95%) | 36 (80%) | 0.928 | 1.06 | [0.29-3.94] |
| **Behaviour** ^1^ | | | | | | |
|  | | (n=20) | (n=43) |  |  |  |
| Non-stricturing,  Non-penetrat. (B1) | | 7 (35%) | 6 (14%) | 0.118 | 2.52 | [0.79-8.02] |
| Stricturing (B2) | | 5 (25%) | 10 (23%) | 0.544 | 0.69 | [0.21-2.28] |
| Penetrating (B3) | | 8 (40%) | 27 (63%) | **0.043** | 0.32 | [0.11-0.97] |
| **Use of immunosuppressive agents** ^2^ | | | | | | |
|  | | (n=21) | (n=42) |  |  |  |
|  | | 16 (80%) | 40 (95%) | 0.120 | 0.32 | [0.08-1.35] |
| **Surgery because of CD** ^3^ | | | | | | |
|  | | (n=21) | (n=41) |  |  |  |
|  | | 12 (57%) | 28 (68%) | 0.287 | 0.55 | [0.18-1.65] |
| **Fistulas** | | | | | | |
|  | | (n=20) | (n=43) |  |  |  |
|  | | 8 (40%) | 27 (62.8%) | **0.043** | 0.32 | [0.11-0.97] |
| **Perianal fistulas** | | | | | | |
|  | | 2/20 (10%) | 18/43 (44%) | **0.016** | 0.14 | [0.03-0.69] |
| **Stenosis** | | | | | | |
|  | | (n=20) | (n=39) |  |  |  |
|  | | 12 (60%) | 25 (64%) | 0.913 | 1.06 | [0.36-3.16] |

**Supplemental table S6.** Phenotype stratified by genotype in CD patients carrying the SNP rs72796353 plus one of the main *NOD2* mutations (AC/+NOD2) and in CD patients carrying the SNP rs72796353 without one of the main *NOD2* mutations (AC/-NOD2). For each variable, the number of patients included is given. ^1^Disease behaviour was defined according to the Montreal classification [27]. A stricturing disease phenotype was defined as presence of stenosis without penetrating disease. The diagnosis of stenosis was made surgically, endoscopically, or radiologically (using MR enteroclysis).^2^ Immunosuppressive agents included azathioprine, 6-mercaptopurine, methotrexate, infliximab, and/or adalimumab.^3^ Only surgery related to CD-specific problems (e.g., ileocecal resection, fistulectomy, colectomy, ileostomy) was included.
