# Supplementary material for: The NOD2 Single Nucleotide Polymorphism rs72796353 (IVS4+10 A>C) Is a Predictor for Perianal Fistulas in Patients with Crohn's Disease in the Absence of Other NOD2 Mutations
Source: PLoS One. 2015 Jul 6;10(7):e0116044. doi: 10.1371/journal.pone.0116044 (PMC4493062; doi:10.1371/journal.pone.0116044)
Supplement: S7 Table — C allele frequencies in patients with CD were similar to the data reported by the HAPMAP project. Interestingly, they were significantly different compared to those published by Lesage et al. [16] and Tukel et al. [24] In the French study, rs72796353 was not found in controls, whereas Tukel et al. [24] observed significant lower allele frequencies in Jewish families compared to our German population. (DOC) [file pone.0116044.s007.doc]

|  | **Crohn’s disease** | **Controls** | **p value** |
| --- | --- | --- | --- |
| **Schnitzler et al. (own cohort)** | 3.22 | 2.78 | 0.612 |
| **Lesage et al.^10^** | 0.77 | 0.00 | 0.090 |
| **Tukel et al. ^20^** | 1.18 | 0.83 | 0.821 |
| **HAPMAP** | N/A | 2.26 | N/A |

**Supplemental table S7.** Comparison of the observed allele frequencies of rs72796353 with data published in the literature (Fishers exact test). C allele frequencies in patients with CD were similar to the data reported by the HAPMAP project. Interestingly, they were significantly different compared to those published by Lesage et al. [16] and Tukel et al. [24] In the French study, rs72796353 was not found in controls, whereas Tukel et al. [24] observed significant lower allele frequencies in Jewish families compared to our German population.
